# Draft Genome Sequence of the Tumor-Targeting *Salmonella enterica* Serovar Typhimurium Strain SL7207

**DOI:** 10.1128/genomeA.01591-16

**Published:** 2017-02-02

**Authors:** Síle A. Johnson, Michael J. Ormsby, Daniel M. Wall

**Affiliations:** Institute of Infection, Immunity and Inflammation, College of Medical, Veterinary and Life Sciences, University of Glasgow, Glasgow, United Kingdom

## Abstract

*Salmonella enterica* serovar Typhimurium strain SL7207 is a genetically modified derivative of strain SL1344, which preferentially accumulates in tumors and can be used as a vehicle for tissue-specific gene delivery *in vivo*. Here, we report the draft genome sequence of SL7207, confirming a purported *aroA* deletion and four single-nucleotide polymorphisms compared to SL1344.

## GENOME ANNOUNCEMENT

*Salmonella enterica* serovar Typhimurium is most well known for causing acute gastroenteritis. If left untreated, S. Typhimurium infection can lead to systemic disease resulting in host septic shock and, in extreme cases, death. Despite these adverse effects, multiple *Salmonella* serovars have been demonstrated to preferentially colonize tumor tissue when administered intravenously and delay tumor growth. SL7207 is one of the most promising strains utilized for this purpose (1). SL7207 is reported to have a deletion in *aroA*, resulting in bacterial auxotrophy for two compounds: ρ-aminobenzoic acid and 2,3-dihydroxybenzoate (2). These compounds are not found in mammalian tissue, rendering the bacteria attenuated in the mammalian host and lending to its suitability as a therapeutic agent. The parent strain of SL7207, SL3261, was engineered as a vaccine strain, and it has been used in cancer therapy studies (1, 3, 4). However, many studies have identified the tumor-preferential localization of SL7207 when administered intravenously (5–7). Multiple studies have employed SL7207 for its tumor disruption and tumor-specific DNA vaccine delivery, as well as identified tumor-specific *Salmonella* promoters and immune components involved in *Salmonella*-tumor localization. Furthermore, bioluminescent SL7207 has been employed as an agent to identify the location of tumors and metastasis *in vivo* (8, 9).

However, a detailed analysis of the genetic makeup of this strain is lacking. To gain a deeper understanding of the mechanisms underlying the antitumor properties of SL7207, its entire genome was sequenced.

Genomic DNA of S. Typhimurium SL7207 was extracted from a freshly grown single colony using an Illumina Nextera XT DNA sample kit per the manufacturer’s protocol (Illumina, USA). Sequencing was performed by Illumina MiSeq using a 2 × 250 paired-end protocol. Read quality analysis and trimming were conducted using Trimmomatic and then quality assessed using in-house scripts combined with SAMtools, BedTools, and BWA-MEM. *De novo* assembly was conducted with SPAdes version 3.5, resulting in a total of 68 contigs, with 30 larger than 1,000 bp. The draft genome of strain SL7207 contains 5,026,283 bp, with 52.16% G+C content, and encodes 4,754 coding sequences (CDSs), nine rRNAs, and 84 tRNAs. S. Typhimurium SL1344, in comparison, encodes 4,605 CDSs, 22 rRNAs, and 85 tRNAs. The contigs of SL7207 were reordered against the complete genome of SL1344, and aligned using progressiveMauve (version 20150226, build 10) (10). A list of single-nucleotide polymorphisms (SNPs) was generated from Mauve and visually inspected using CLC Genomics Workbench version 7.

The seminal differentiating feature between the ancestral virulent strain, SL1344, and SL7207 is the 1,194-bp deletion in *aroA* in SL7207. Furthermore, eight SNPs were identified: four intergenic and four intragenic, with two being synonymous and two being nonsynonymous. SNPs were found in four genes; SNPs in *menC* (menaquinone metabolism) (11) and *ackA* (acetyl-CoA biosynthesis) (12) were synonymous, whereas SNPs in *ptsL* (mannose transport) (13, 14) and *ycdT* (motility) (15) were nonsynonymous.

The genome sequence of SL7207 provides the foundation for further molecular characterization of the strain in its use as a therapeutic agent in the treatment of cancer.

### Accession number(s).

This draft genome project has been deposited at DDBJ/EMBL/GenBank under the accession number MPJV00000000 (BioProject PRJNA350897; BioSample SAMN05949357). The version described in this paper is the first version, MPJV01000000.
